# Investigating the Agreement Between Cardiovascular Disease Risk Calculators Among People Diagnosed With Schizophrenia

**DOI:** 10.3389/fpsyt.2018.00685

**Published:** 2018-12-12

**Authors:** Alexandra Berry, Richard J. Drake, Roger T. Webb, Darren M. Ashcroft, Matthew J. Carr, Alison R. Yung

**Affiliations:** ^1^Division of Psychology & Mental Health, School of Health Sciences, Faculty of Biology, Medicine and Health, Manchester Academic Health Sciences Centre (MAHSC), University of Manchester, Manchester, United Kingdom; ^2^Greater Manchester Mental Health NHS Foundation Trust, Manchester, United Kingdom; ^3^Division of Pharmacy & Optometry, School of Health Sciences, Faculty of Biology, Medicine and Health, Manchester Academic Health Sciences Centre (MAHSC), University of Manchester, Manchester, United Kingdom; ^4^Orygen, The National Centre of Excellence in Youth Mental Health, The University of Melbourne, Parkville, VIC, Australia

**Keywords:** schizophrenia, cardiovascular risk, risk calculator, cardiovascular disease, risk score

## Abstract

**Background:** People diagnosed with schizophrenia have a much reduced life expectancy compared to the general population, and a more than doubled risk of dying from cardiovascular disease (CVD). Existing CVD risk calculators can be used to detect people with an elevated predicted risk of CVD to inform interventions to reduce risk.

**Aims:** This study aimed to compare four different risk calculators for 10-year predicted CVD risk in a sample of people with schizophrenia.

**Methods:** Thirty participants with a diagnosis of schizophrenia spectrum disorders living within Greater Manchester, United Kingdom took part. Ten-year predicted cardiovascular risk scores were calculated using four different models: QRISK3, Framingham, PRIMROSE BMI, and PRIMROSE lipid. Risk estimates and classified risk categories were compared.

**Results:** QRISK3 identified 11 (39%) as having >10% risk of a CV event within 10 years, 4 (14%) of whom exceeded 20%. The Framingham model identified 4 (14%) as exceeding 10%, none of whom exceeded 20%. PRIMROSE risk calculators identified no participants as having >10% risk of a CV event within 10 years. Pairwise concordance correlation coefficients between types of model ranged 0.22–0.77. Mean (± SD) age was 40 (± 10) years but QRISK3's mean “Heart age” was 58 (± 14) years.

**Conclusion:** Risk calculators generate differing predicted CVD risk scores for patients with schizophrenia. Using one risk calculator might yield different recommended monitoring and treatment plans compared to another. Clinicians should therefore take into account other patient-related factors, such as patients' preferences and other underlying physical conditions when making treatment decisions.

## Introduction

Globally, people diagnosed with schizophrenia have a mortality rate 2–3 times that of the general population (1) and their life expectancy is reduced by 10 to 30 years (1–3). As in the general population, cardiovascular disease (CVD) is the most common cause of death among these individuals (4–6). A meta-analysis of over 3 million patients with severe mental illness (SMI) and over 113 million controls from 92 studies with a cross-sectional or retrospective/prospective longitudinal design showed that people with schizophrenia are at more than double the risk of dying from CVD and are at 59% greater risk of developing coronary heart disease (CHD) compared to the remainder of the population (7).

CVD risk calculators are used to estimate an individual's absolute risk of having a heart attack or stroke within a specified amount of time, such as 5 or 10 years. These calculators have been developed in large population studies and their validity and reproducibility assessed using population-level statistics that measure calibration and discrimination (8–11). CVD risk prediction scores are used in clinical practice with the general population to guide whether early intervention could be beneficial, such as prescribing medication to lower blood pressure and cholesterol (12). Moreover, in the United Kingdom (UK), the National Institute for Health and Care Excellence (NICE), which issues national clinical guidelines in the UK to improve health and social care (13) recommend routine monitoring of cardiovascular risk in people with schizophrenia (14).

Risk prediction scores can also be used as surrogate endpoints for intervention studies; that is, an outcome in a clinical trial would be change in CVD risk score after a certain period of time (15). In the schizophrenia population, risk prediction scores have been used as surrogate endpoints in intervention studies, for example the Clinical Antipsychotic Trials of Intervention Effectiveness (CATIE) trial included 1,125 people with schizophrenia and compared change in predicted 10-year CHD risk using the Framingham risk score (16) during antipsychotic treatment.

There are currently more than 360 CVD risk calculators available (17). A previously published review reported that 25 different calculators allocated the same person to a different risk category 33% of the time (18). Given the differences between risk calculators when applied in the general population, it would be useful to know whether or not consistency is similarly poor when the calculators are applied specifically to people with schizophrenia. Therefore, the aim of this study was to compare the 10-year absolute CVD risk score in a sample of 30 people with schizophrenia using four different risk calculators: QRISK3 (9), Framingham (11), The PRedIction, and Management of cardiovascular Risk in peOple with SEvere mental illnesses (PRIMROSE) body mass index (BMI) and PRIMROSE lipid (10). Whilst there are a large number of calculators to select from, we decided to focus on risk calculators that include risk factors applicable to people with schizophrenia, such as an SMI diagnosis and antipsychotic use. Such risk factors may confer additional risk for CVD, independent of traditional predictors (19, 20). Furthermore, we chose to assess Framingham due to it being a calculator still commonly used worldwide, including in this patient group. Table 1 shows a comparison of risk factors measured in each model that we have selected: QRISK3, Framingham, PRIMROSE BMI, and PRIMROSE Lipid.

**Table 1:** Comparison of risk factors included in four cardiovascular risk calculators.

**Variables**	**Cardiovascular risk calculators**			
	**Framingham**	**QRISK3**	**PRIMROSE Lipid**	**PRIMROSE BMI**
Age	✓	✓	✓	✓
Gender	✓	✓	✓	✓
Ethnicity	✗	✓	✗	✗
HDL cholesterol	✓	✓	✓	✗
Total cholesterol	✓	✓	✓	✗
Systolic blood pressure	✓	✓	✓	✓
SD of two systolic blood pressure readings	✗	✓	✗	✗
Type 2 diabetes	✓	✓	✓	✓
Type 1 diabetes	✗	✓	✗	✗
Current smoker	✓	✓	✓	✓
Ex-smoker	✗	✓	✓	✓
Light/moderate/heavy smoker	✗	✓	✗	✗
Hypertension treatment	✓	✓	✓	✓
BMI	✗	✓	✗	✓
Townsend index of deprivation	✗	✓	✓	✓
Family history of CVD	✗	✓	✗	✗
Chronic kidney disease	✗	✓	✗	✗
Migraines	✗	✓	✗	✗
Lupus (SLE)	✗	✓	✗	✗
Rheumatoid arthritis	✗	✓	✗	✗
Erectile dysfunction	✗	✓	✗	✗
Atrial fibrillation	✗	✓	✗	✗
History of heavy drinking	✗	✗	✓	✓
SMI diagnosis	✗	✓	✓	✓
Prescription for atypical antipsychotics	✗	✓	✓	✓
Prescription for typical antipsychotics	✗	✗	✗	✓
Prescription for antidepressants	✗	✗	✓	✓
Oral steroids	✗	✓	✗	✗

## Methods

### Sample

Individuals were eligible to take part if they were aged 18–65 and had a diagnosis of schizophrenia or related psychosis, according to the ICD-10 classification system (21). Individuals were excluded if they were non-English speaking, were unable to give their informed consent, or they had a history of CVD as specified by the CVD risk calculators used (9, 11), as well as NICE guidelines (22). These individuals are already considered at high-risk of CVD events, therefore generating a risk score does not add useful additional information (22). Participants were recruited from Greater Manchester Mental Health NHS Foundation Trust services in England. All were given an information sheet to read and provided their written informed consent. The study received ethical approval from the North West Research Ethics Committee (17/NW/0368). Thirty participants were included in the study. Seventeen participants resided in the community and 13 were inpatients on mental health or rehabilitation wards. Participants' histories of diagnosed CVD were established by reviewing their medical records for related diagnoses and for any prescribed medication associated with cardiometabolic conditions.

### CVD Risk Calculators

#### Framingham

The Framingham risk scores (11) apply an algorithm that calculates the predicted CVD risk from risk factors such as diabetes, dyslipidemia, hypertension (23–25), smoking, and obesity (26, 27). This risk calculator is frequently used in the general population but has also been applied to population groups with SMI (28). The prevalence of these risk factors is considerably raised in people with schizophrenia (4, 29–31) and people with schizophrenia have been shown to have higher risk scores than the general population (32–34). However, people with SMI were excluded from the Framingham cohort. As such, risk factors of particular relevance to those with an SMI diagnosis, such as antipsychotic use are not included. Yet antipsychotic use can cause significant morbidity, including weight gain (35, 36) and diabetes (37), conditions that are associated with an increased risk of CVD.

When applying the Framingham risk calculator to patients in the United Kingdom (UK), NICE recommends using the modified version (38). The modified version entails multiplying the results of the US Framingham score by 1.4 for south Asian men in the UK. Patients are considered to be at high risk of a CVD event over the next 10 years if they score 20% or greater (11). Estimates of risk include several CVD outcomes, including coronary death, myocardial infarction, coronary insufficiency, angina pectoris, ischaemic stroke, haemorrhagic stroke, transient ischaemic attack, peripheral artery disease, and heart failure (39).

#### PRIMROSE

The PRIMROSE BMI and lipid models are 10-year predicted cardiovascular risk calculators specifically designed for people with SMI (10). They include risk factors relevant to people with SMI, such as prescription of antipsychotic medication, antidepressant medication, history of heavy alcohol consumption, area-level Townsend index of deprivation (in quintiles) (40) and diagnosis of an SMI. These models were validated using The Health Improvement Network (THIN) UK primary care database (10).

The PRIMROSE BMI risk calculator excludes lipid profile. Patients are considered to be at high risk of a CVD event over the next 10 years if they score 20% or greater (10). The PRIMROSE lipid model excludes BMI and, again, patients are considered to be at high risk of a CVD event over the next 10 years if they score 20% or greater (10). Outcomes for which risks are in predicted in both PRIMROSE risk calculators cover both fatal and non-fatal cardiovascular events including myocardial infarction, angina pectoris, coronary heart disease, major coronary surgery and revascularization, cerebrovascular accident, and transient ischaemic attack (10).

#### QRISK3

The QRISK2 risk calculator is recommended for use with the UK general population by NICE (41). NICE also recommends QRISK2 for use in those with medical conditions including SMI, whilst acknowledging it may underestimate risk in such groups (22). QRISK2 is about to be superseded by QRISK3, which includes both typical risk indicators and specific factors for SMI (i.e., an SMI diagnosis and antipsychotic use) in patients aged 25–84 years (9) and may enable more accurate assessment. The QRISK algorithms have been validated using UK primary care databases including the Clinical Practice Research Datalink (CPRD) (42) and THIN (43–45). As well as calculating the 10-year risk of CVD, QRISK3 also estimates a “heart age.” This is calculated by comparing the ideal age of healthy individuals who receive the same predicted CVD risk score and have the same sex and ethnicity (46). A heart age older than the current age suggests increased risk that is modifiable (46). It is important to emphasize that this is a relative measure and heart age has been found on average to be older than chronological age in every age strata (47). It may be a useful tool to encourage lifestyle modification rather than to inform medication recommendations (48).

Using the QRISK3, patients are considered to be at high risk of a CVD event over the next 10 years if they score 10% or greater (9). This threshold was set following health economics modeling for when to offer medical intervention, such as with statins prescription (38). Risks are estimated for the following CVD outcomes: transient ischaemic attack and related syndromes, angina pectoris, myocardial infarction, subsequent myocardial infarction, complications after myocardial infarction, other acute ischaemic heart disease, chronic ischaemic heart disease, cerebral infarction, and stroke not specified as hemorrhage or infarction (9).

### Anthropometric Measurements

BMI was calculated as weight/height2 (kg/m2). Participants were classified according to the World Health Organization (WHO) criteria as underweight (BMI < 18.5), normal (BMI 18.5–24.99), overweight (BMI 25–29.99) obese (BMI 30–39.99), or severe obesity (BMI ≥40) (49). Medical records were reviewed for any diagnosed physical and mental health conditions, prescribed medication and to confirm self-reported demographic information.

### Statistical Analyses

Data were analyzed using STATA (version 11; Statacorp, TX, USA). Descriptive statistics were reported as medians, means, standard deviations (SDs) and percentages. 10-year predicted cardiovascular risk scores were calculated using published algorithms (9–11). The relationship between each CVD risk calculator model was assessed using concordance correlation coefficients (P_c_). This correlation measures how far the best-fit line differs from the line y = x, by adjusting the *r*-value using a bias correction factor. P_c_ therefore provides a measure of correlation and agreement. Bland-Altman plots were generated to plot the difference against mean between pairs of CVD risk calculators.

## Results

### Sociodemographic and Summary Data

The study sample included 30 participants. Their mean age was 40.4 ± 10.2 years and 83% were male. Most of the participants were overweight, with 33% being either obese (BMI ≥30 kg/m2) or severely obese (BMI ≥40 kg/m2) and the mean BMI was in the overweight range (29.9 ± 7.9; Table 2). Atypical antipsychotics were prescribed to 83% of participants; typical antipsychotics were prescribed to 27% of participants. Both atypical and typical antipsychotics were prescribed to 10% of these participants.

**Table 2:** Distribution of sociodemographic and clinical factors in the sample of patients diagnosed with schizophrenia.

	***N***	**%**	**Mean ± SD**	**Median**
**GENDER**				
Male	25	83		
Female	5	17		
**Age**			40.4 ± 10.2	40
18–34	9	30		
35–64	21	70		
**ETHNICITY**				
White British	19	63		
Black British/African	4	13		
Black British/Caribbean	2	7		
Other ethnic groups	5	17		
**SMOKING**				
Current smoker	22	73		
Ex-smoker	5	17		
Never smoked	3	10		
**DIAGNOSIS OF TYPE 2 DIABETES**				
Yes	4	13		
No	26	87		
**PRESCRIBED ANTI-DEPRESSANT MEDICATION**				
Yes	12	40		
No	18	60		
**PRESCRIBED STATINS**				
Yes	7	23		
No	23	77		
**PRESCRIBED HYPERTENSIVE TREATMENT**				
Yes	7	23		
No	23	77		
**Systolic blood pressure**			125.9 ± 16.7	125.5
< 120 mmHg	11	37		
≥120–139 mmHg	12	40		
≥140 mmHg	7	23		
**BMI**			29.9 ± 7.9	27.5
Underweight (< 18.5)	0	0		
Normal (18.5–24.99)	8	27		
Overweight (25–29.99)	12	40		
Obese (30–39.99)	7	23		
Severe obesity (≥40)	3	10		

*Systolic Blood Pressure*.

### 10-Year Predicted CVD Risk

Participants' absolute 10-year risk of CVD was calculated using four different risk calculators: QRISK3, Framingham, PRIMROSE BMI, and PRIMROSE lipid. Two participants were excluded from the QRISK3 analysis due to their age being below 25 years. One participant was excluded from the Framingham and PRIMROSE lipid analyses due to an inability to draw blood and obtain their lipid profile. QRISK3 identified 11 participants (39%) who were classified into the high-risk category of having a CVD event within 10 years (range 0.4–38.5%), based on a 10% threshold for high-risk. No participants were classified into the high risk category for the PRIMROSE lipid (range 0.1–8.5%), PRIMROSE BMI (range 0.2–7.4%), or Framingham (range 0.1–15.6%) risk calculators, based on a 20% threshold for high-risk. However, if a 10% threshold is imposed across all four calculators, 39% of patients are categorized as high-risk with QRISK3, 14% with Framingham and none with PRIMROSE risk calculators. If a 20% threshold is imposed across all four calculators, 14% are categorized as high risk by QRISK3 and none with other the other risk calculators (Table 3).

**Table 3:** Ten-year predicted CVD risk scores according to 4 different risk calculators.

	**<10%**		**10–19%**		**20% or more**		Mean^*^± SD
	***N***	**%**	***N***	**%**	***N***	**%**	
QRISK3	17	61	7	25	4	14	9.3 ± 8.8
Framingham	25	86	4	14	0	0	4.1 ± 4.1
PRIMROSE Lipid	29	100	0	0	0	0	2.0 ± 2.0
PRIMROSE BMI	30	100	0	0	0	0	2.9 ± 2.1

* *Mean % of 10-year predicted CVD risk*.

### Heart Age

**Figure 1:**
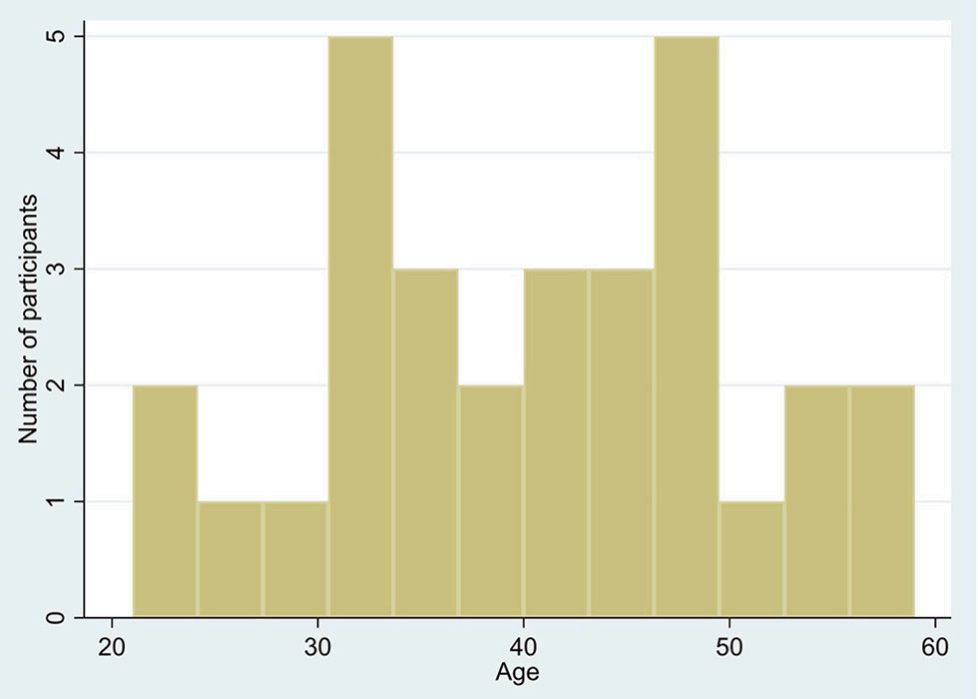
Distribution of age.

**Figure 2:**
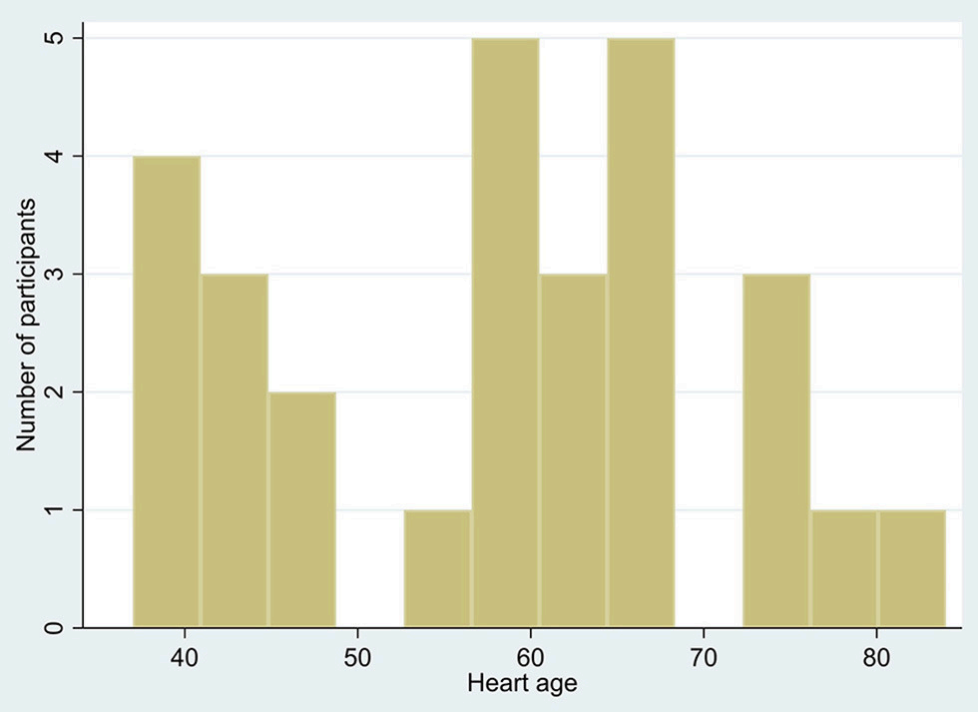
Distribution of heart age.

**Figure 3:**
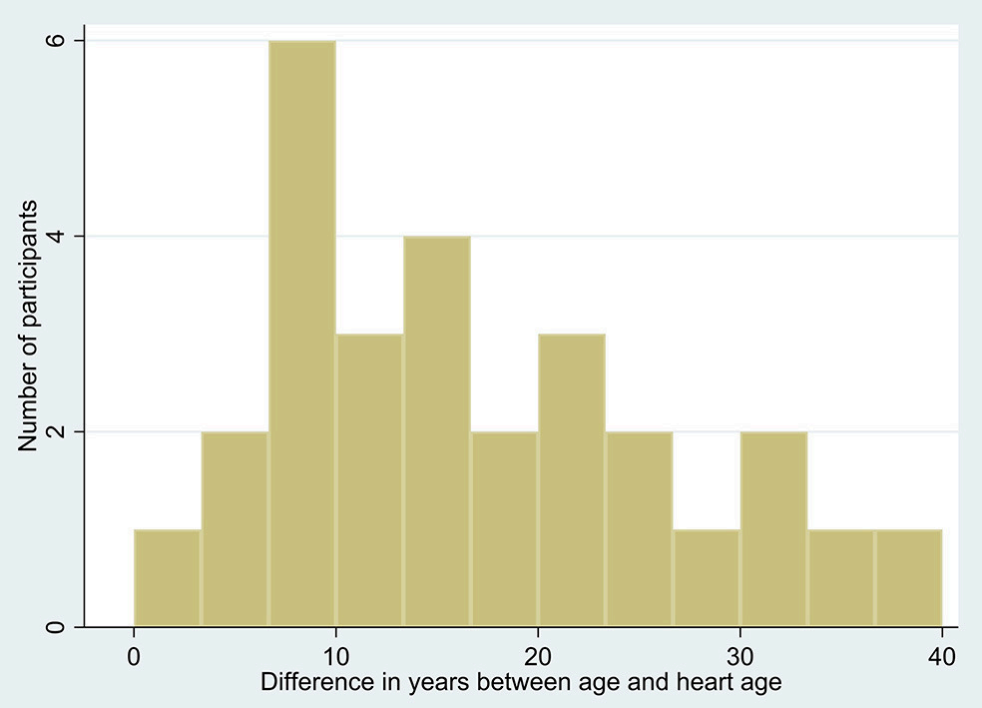
Difference between age and heart age.

Frequency distributions of age and heart age calculated using QRISK3 can be seen in Figures 1, 2. The mean heart age was 58.4 ± 13.5. The mean difference between age and calculated heart age was found to be over 16 years with one individual scoring a heart age 40 years above their actual age (Figure 3).

### Agreement Between 10-Year Predicted CVD Risk Models

**Figure 4:**
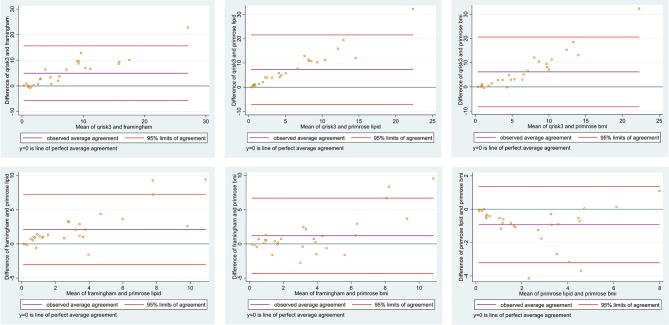
Difference against mean between 10-year predicted CVD risk calculators.

Pairwise concordance correlation coefficients were highest between the two PRIMROSE measures but the QRISK3 and PRIMROSE metrics had notably poor correlations, not even reaching the acceptable range at their upper CIs and close to zero at the lower CIs (Table 4; Figure 4).

**Table 4:** Agreement among 10-year predicted CVD risk models (%).

	**Difference**		**95% Limits of agreement**	**P_**c**_ (95% CI)**
	**Mean**	**SD**		
QRISK3 vs. Framingham	5.0	5.4	−5.7 to 15.7	0.549 (0.41 to 0.69)
QRISK3 vs. PRIMROSE Lipid	7.2	7.3	−7.1 to 21.6	0.224 (0.12 to 0.32)
QRISK3 vs. PRIMROSE BMI	6.2	7.4	−8.2 to 20.6	0.226 (0.12 to 0.33)
Framingham vs. PRIMROSE Lipid	2.1	2.6	−3.0 to 7.3	0.543 (0.38 to 0.70)
Framingham vs. PRIMROSE BMI	1.2	2.8	−4.3 to 6.7	0.581 (0.41 to 0.75)
PRIMROSE Lipid vs. PRIMROSE BMI	−0.9	1.2	−3.2 to 1.4	0.767 (0.63 to 0.91)

*P_c_, Concordance correlation coefficient; CI, confidence intervals*.

## Discussion

We applied four risk calculators to determine the predicted CVD risk for 30 patients with schizophrenia over the next 10 years. Mean QRISK3 calculated risk estimates were approximately 4 times higher than the PRIMROSE lipid calculated risk estimates, and roughly 3 times higher than PRIMROSE BMI. Moreover, agreement between calculators was poorest between QRISK3 and the PRIMROSE calculators, despite these calculators taking into account diagnosis of SMI and use of antipsychotic medication within their algorithms. These risk factors are known to have a complex effect on cardiovascular risk (9) and the lack of agreement highlights the variability in the weighting of risk factors between these models, which impacts the relative risk. Agreement between Framingham and the three other risk calculators was more acceptable but not high. People with SMI were excluded from the Framingham heart study cohort and previous research comparing cardiovascular risk calculators in the general population has recommended selecting calculators that have been developed from a population similar to the patient (50).

People with schizophrenia and related conditions are recommended to attend for regular physical health assessments, including checking for cardiometabolic indicators specifically so that clinicians may offer any required interventions (51). For the purposes of CVD prevention, it is therefore important for clinicians to be able to identify individuals at highest risk of developing CVD. There is currently a lack of reliable evidence indicating the superiority of one CVD risk calculator over another, particularly in relation to persons with SMI. Our data suggest that there are important differences between calculators.

The threshold for classifying a patient as high-risk of having a CVD event over the next 10 years differed between the models. Thirty nine percent of participants were classified as high-risk using QRISK3, but all were classified as low-risk using the other calculators. Framingham and the PRIMROSE risk calculators both set the threshold as 20% or greater, while QRISK3 sets the threshold as 10% or greater. NICE guidelines recommend using a 10% threshold for initiating preventative therapy with statins (52). It is however not clear whether this is the optimal threshold for modifying risk in people with schizophrenia. For clinicians it is therefore not clear when to initiate preventative therapy or which risk calculator to use with patients with SMI. NICE emphasizes that faced with the challenge of applying population-based measures to individuals, clinicians should exercise their judgement and include patients' preferences when making clinical decisions in relation to CVD risk scores (53). There may also be other patient-related factors that should be considered, such as socioeconomic status or other underlying medical conditions (53).

The risk factors included in the algorithms for CVD risk calculators typically include age, gender, medical diagnoses, prescription to medications, BMI, blood pressure, and lipid profile. With the exception of smoking in all risk calculators, and history of heavy drinking within PRIMROSE risk calculators (10), lifestyle screening is not included. Lifestyle screening contributes little to CVD risk calculator algorithms, partly because resource limitations mean lifestyle factors are not routinely recorded within primary care databases from which risk models derive. However, many lifestyle factors have been found to increase the risk of CVD both in the general population and SMI groups. Meta-analyses show that sedentary behavior in the general population is associated with increased risks of diabetes, CVD and cardiovascular and all-cause mortality (54); and that those with schizophrenia group engage in high levels of sedentary behavior (55). Furthermore, sleep disorders are reported in 30–80% of people with schizophrenia (56, 57). They take longer to fall asleep, sleep longer, take daytime naps and have severely fragmented sleep compared to healthy controls (58). There is emerging evidence that disrupting sleep can cause health problems, such as CVD (59). It may be that sleep quality needs to be routinely enquired about and its role in CVD risk calculators investigated

Comparing models, it is notable that the low PRIMROSE and Framingham risk scores seem at odds with clinical impressions of participants and with studies of life expectancy, CVD and CVD risk factor prevalence in people with schizophrenia. People with schizophrenia have 2.5 times the risk of dying compared to the general population (1), and a mean age of death from CVD in schizophrenia patients was found to be 51 years (60). Moreover, general population life expectancy in the UK is lowest in northern urban England, including Manchester (61), with healthy male life expectancy at birth in Greater Manchester in the 50s (54). This implies that a substantial proportion of this sample should be in a high-risk category. QRISK3 estimated our participant heart ages to be a mean of 16 years older than chronological age. Whilst “heart age” may not be an accurate enough tool to inform medical decision-making (48), an age adjustment of this size may be similar to the substantial reduction in life expectancy in this group.

Our study was limited by its small sample size, which makes it difficult to draw generalisable conclusions and we therefore recommend repeating this study on a considerably large scale. The sample was recruited from Greater Manchester in England by volunteers. This is an area where health and life expectancy is unusually poor in the general population (54), so this sample may well not be representative. However, this may have enabled us to detect higher rates of predicted CVD risk in this group. A longitudinal study of a representative cohort of sufficient size to accurately estimate agreement among CVD risk calculators is indicated. This study underlines the importance of such work in that, even in a small study, differences were observed between algorithms in their risk predictions.

## Conclusions

Existing CVD risk calculators have low agreement with each other in our sample of people diagnosed with schizophrenia. This makes it challenging to draw conclusions about which tool is most suitable for clinicians to use with this population group, and how to treat patients who fall in different categories when using one calculator rather than another. It is recommended that clinicians consider multiple patient-related factors in addition to predicted CVD risk score when recommending treatment. Furthermore, participants in our study had a calculated heart age that was much older than their actual ages. Of interest would be whether adjusting the age of patients with schizophrenia more accurately predicts CVD events. Further research is needed with larger numbers and with longitudinal follow-up to assess actual CVD events to understand this further. Also of interest would be the use of large datasets to examine the question of CVD risk in schizophrenia. Such studies should also investigate if any of the factors known to increase risk of CVD in the general population that are also common in schizophrenia, such as physical inactivity (55–57), high rates of sedentary behavior (58), and sleep disturbance (59, 62) impact on CVD risk, independent of the risk factors already included in existing CVD risk calculators.

## Author Contributions

AB, AY, and RD contributed conception and design of the study. AB collected the data. AB and RD performed the statistical analysis. AB and AY wrote the first draft of the manuscript. AB, AY, and RD wrote sections of the manuscript. All authors contributed to manuscript revision, read and approved the submitted version.

### Conflict of Interest Statement

AB published a research paper with the editor in 2016: How much physical activity do people with schizophrenia engage in A systematic review, comparative meta-analysis and meta-regression. The remaining authors declare that the research was conducted in the absence of any commercial or financial relationships that could be construed as a potential conflict of interest.
